# Spectral decomposition of EEG microstates in post-traumatic stress disorder

**DOI:** 10.1016/j.nicl.2022.103135

**Published:** 2022-07-29

**Authors:** Braeden A. Terpou, Saurabh B. Shaw, Jean Théberge, Victor Férat, Christoph M. Michel, Margaret C. McKinnon, Ruth A. Lanius, Tomas Ros

**Affiliations:** a Department of Psychiatry and Behavioural Neurosciences, McMaster University, Hamilton, Canada; b Mood Disorders Program, St. Joseph’s Healthcare Hamilton, Hamilton, Canada; c Department of Psychiatry, Western University, London, Canada; d Department of Medical Biophysics, Western University, London, Canada; e Imaging Division, Lawson Health Research Institute, London, Canada; f Homewood Research Institute, Guelph, Canada; g Vector Institute, Toronto, Canada; h Department of Basic Neurosciences, University of Geneva, Geneva, Switzerland; i Centre for Biomedical Imaging (CIBM), Lausanne-Geneva, Switzerland

**Keywords:** EEG, Microstates, Resting-state, Machine learning, PTSD

## Abstract

Microstates offer a promising framework to study fast-scale brain dynamics in the resting-state electroencephalogram (EEG). However, microstate dynamics have yet to be investigated in post-traumatic stress disorder (PTSD), despite research demonstrating resting-state alterations in PTSD. We performed microstate-based segmentation of resting-state EEG in a clinical population of participants with PTSD (N = 61) and a non-traumatized, healthy control group (N = 61). Microstate-based measures (i.e., occurrence, mean duration, time coverage) were compared group-wise using broadband (1–30 Hz) and frequency-specific (i.e., delta, theta, alpha, beta bands) decompositions. In the broadband comparisons, the centro-posterior maximum microstate (map E) occurred significantly less frequently (*d* = -0.64, *p*FWE = 0.03) and had a significantly shorter mean duration in participants with PTSD as compared to controls (*d* = -0.71, *p*FWE < 0.01). These differences were reflected in the narrow frequency bands as well, with lower frequency bands like delta (*d* = -0.78, *p*FWE < 0.01), theta (*d* = -0.74, *p*FWE = 0.01), and alpha (*d* = -0.65, *p*FWE = 0.02) repeating these group-level trends, only with larger effect sizes. Interestingly, a support vector machine classification analysis comparing broadband and frequency-specific measures revealed that models containing only alpha band features significantly out-perform broadband models. When classifying PTSD, the classification accuracy was 76 % and 65 % for the alpha band and the broadband model, respectively (*p* = 0.03). Taken together, we provide original evidence supporting the clinical utility of microstates as diagnostic markers of PTSD and demonstrate that filtering EEG into distinct frequency bands significantly improves microstate-based classification of a psychiatric disorder.

## Highlights

• EEG microstates reveal significant temporal differences in PTSD.
• Microstate E (with centro-posterior maximum) is temporally underrepresented in PTSD.
• In PTSD, microstate E has a reduced occurrence and a shorter mean duration.
• Spectral decomposition of EEG microstates improves microstate-based classification.
• Alpha band SVM features yield the highest classification accuracy of PTSD (76%).

## 1 Introduction

When asked to simply let the mind wander, individuals commonly find themselves drawn toward thinking about the past, planning ahead, or engaging in a variety of self- and other-directed processing (Smallwood & Schooler, 2006). These multi-dimensional, internally-directed ways of thinking occupy what has been referred to as the resting-state. Using fMRI, researchers have discovered a variety of large-scale, resting-state networks (RSNs) to be active at rest (Smitha et al., 2017). However, fMRI records brain activity on a multi-second time scale, resulting in a temporally ‘smeared’ signal that makes it difficult to assess fast-scale brain dynamics. In order to respond to an environment with rapidly changing demands, brain networks must adaptively reorganize into distinct spatial patterns on a sub-second time scale (Bressler, 1995). Electroencephalography (EEG) records changes in electrical activity on a millisecond time scale, making it well-suited to assess fast-scale brain dynamics. However, common EEG analyses based on the Fourier transform filter EEG signals into frequency bands and average them across multi-second time windows, losing considerable sensitivity in the time domain. In response to the above, EEG microstates have emerged as a promising framework to study whole-brain, temporal dynamics – an approach that has been used increasingly in the clinical literature as of late.

The momentary scalp EEG field reflects the transient global state of the brain. It represents the summation of all concurrently active sources in the brain irrespective of their frequency (Imperatori et al., 2014). A series of momentary scalp EEG field patterns will remain quasi-stable for periods of about 50–120 ms. During these periods, the topography remains fixed, while the polarity may invert. These quasi-stable periods of fixed topography have been referred to as EEG microstates (Lehmann et al., 1987). Using clustering techniques, researchers have found that a limited number of microstate maps (usually-four (A → D) to seven (A → G)) with prototypical configurations can explain>70 % of the variability in the temporal dynamics (Koenig et al., 1999, Brodbeck et al., 2012). Interestingly, EEG microstates have been found to correlate variously with fMRI-defined RSNs, with Britz and colleagues (2010) and others (Musso et al., 2010, Yuan et al., 2012) finding that microstate maps A and B correlate more strongly with sensory-based, auditory and visual networks, respectively, while microstate maps C and D correlate more strongly with cognitive control and attention networks, respectively.

Critically, fMRI-defined RSNs have been found to be altered significantly in post-traumatic stress disorder (PTSD) (Wang et al., 2016, Akiki et al., 2018). In particular, the default mode network, a RSN mediating self-related processing and autobiographical memory, shows decreased resting-state functional connectivity in PTSD (Bluhm et al., 2009, Lanius et al., 2010, Sripada et al., 2012, Akiki et al., 2017), while the salience network, a RSN mediating bottom-up attention processing, shows increased resting-state functional connectivity in participants with PTSD as compared to healthy controls (Sripada et al., 2012, Thome et al., 2014, Nicholson et al., 2016, Akiki et al., 2018).

Given that fMRI-defined RSNs have been found to be altered in PTSD, and that EEG microstates correlate to these fMRI-defined RSNs (Britz et al., 2010, Musso et al., 2010), we hypothesized that microstate analyses could assist in the discovery of PTSD diagnostic markers (so-called ‘neuromarkers’). Elsewhere, microstate analyses have led to significant clinical insights in schizophrenia (Lehmann et al., 2005, da Cruz et al., 2020), psychosis (de Bock et al., 2020, Murphy et al., 2020a), and major depressive disorder alike (Damborská et al., 2019, Murphy et al., 2020b). However, microstate analyses using the standard microstate map definitions have never been performed in participants with PTSD.

Of note, Yuan et al. (2018) have investigated resting-state microstate dynamics in participants with combat-related PTSD using multimodal imaging, revealing distinct microstate dynamics in maps found to be correlated with fMRI-defined, default mode and salience networks. However, Yuan and colleagues used an independent component analysis to cluster EEG field patterns group-specifically, identifying ten slightly different group-specific microstate maps. Although useful, group-specific microstate maps do not readily permit direct group comparisons. Moreover, since they explored ten microstate maps instead of the typical four to seven, many of the common ‘canonical’ microstate maps were splintered into multiple maps, making it difficult to interpret these findings in the context of the broader literature.

Naturally, traditional EEG-based analyses have been performed in PTSD, namely based on the power spectrum. Power-based analyses (e.g., power spectral density) focus on the frequency domain instead of the time domain, sacrificing temporal sensitivity and producing the so-called ‘time–frequency uncertainty principle.’ In PTSD, increases in theta power over central brain regions, increases in beta power over frontal, central, and occipital brain regions, and decreases in alpha power over frontal, central, and occipital brain regions have been revealed at rest (Begić et al., 2001, Jokić-Begić and Begić, 2003, Todder et al., 2012). These measures constitute the strongest PTSD-linked EEG correlates; particularly the reduced power of the alpha rhythm, which has been replicated independently by our laboratory (Ros et al., 2017) and others (Clancy et al., 2017).

In the present study, we investigated and compared resting-state microstate dynamics (i.e., occurrence, mean duration, time coverage) between participants with PTSD and non-traumatized, healthy controls. We performed microstate-based segmentation two ways: with broadband (1–30 Hz) and frequency-specific (i.e., spectral) EEG. In addition to permutation tests on the group means, we performed machine learning to compare the classification accuracy of broadband and frequency-specific microstate-based models (Férat et al., 2022). Given that participants with PTSD generally display reduced alpha oscillations (Jokić-Begić and Begić, 2003, Ros et al., 2017, Clancy et al., 2017), we hypothesized that the spectral decomposition of EEG microstates would provide greater specificity of these microstate dynamics. Moreover, since alpha oscillations are most prominent over the occipital cortex, we hypothesized that the microstate maps with maximums over posterior and occipital channels would be more likely to display the strongest group differences. Overall, the present work hopes to introduce novel, microstate-based neuromarkers specific to PTSD, a population yet to be explored using standard microstate map definitions.

## 2 Materials and Methods

### 2.1 Participants

In the present study, we pooled two previously collected datasets and re-analyzed them using a microstate framework. The first dataset (Ros et al., 2017) included 20 participants with PTSD (mean age = 39.9 years, SD = 13.7 years, 8 female) and 30 non-traumatized, healthy adults (mean age = 39.4, SD = 8.7 years, 26 female), while the second dataset (Nicholson et al., 2020) included 41 participants with PTSD (mean age = 42.3 years, SD = 12.5 years, 28 female) and 32 non-traumatized, healthy adults (mean age = 42.4 years, SD = 10.7 years, 22 female). In order to have a balanced number of PTSD and control participants, we removed one healthy control who had the poorest EEG signal quality, resulting in a total of 61 participants with PTSD and 61 healthy controls. Both these investigations were approved by the Research Ethics Board at Western University in accordance with the Code of Ethics of the World Medical Association (Declaration of Helsinki) for experiments involving human participants. Participants were recruited via referrals made by family physicians, mental health professionals, and local clinics, as well as by advertisements posted throughout the London, Ontario community.

Participants in the PTSD group had a primary diagnosis of PTSD as determined using the Clinician-Administered PTSD Scale (CAPS; cut-off score > 50; Blake et al., 1995) and the Structured Clinical Interview for DSM-IV Axis-I Disorders (SCID; First, 1997; Weathers et al., 2018) (Table 1). Participants who were recruited originally by Ros and colleagues (2017) were assessed with the CAPS-IV, while participants recruited later by Nicholson and colleagues (2020) were assessed with the CAPS-5. Exclusion criteria included a lifetime diagnosis of a psychotic disorder, bipolar disorder, substance use disorder within the last six months, history of head injury involving loss of consciousness, serious medical illness, pregnancy, and non-compliance with MRI safety standards. In addition to these criteria, Nicholson and colleagues (2020) excluded participants if they had ever been involved in a previous trauma-focused psychotherapy treatment or had ever received neurofeedback therapy. Healthy controls were excluded if they had been diagnosed with any lifetime Axis-I psychiatric disorders or if they were currently taking psychotropic medication. All participants with PTSD who were prescribed psychotropic medication (N = 35) were on a stable dose prior to study involvement. These medications included antidepressants (Total: N = 30; SSRIs: N = 24; SNRIs: N = 3; SARIs: N = 1; Tricyclic: N = 1; Tetracyclic: N = 1), atypical antipsychotics (Total: N = 7), sedatives (Total: N = 12; Benzodiazepines: N = 9; Cyclopyrrolone: N = 4), and stimulants (Methylphenidate: N = 2).

**Table 1:** Demographic and Clinical Measures.

	**PTSD (N = 61)**
AGE	41.51 ± 12.84
SEX	Males = 25, Females = 36
EDUCATION LEVEL	Grade 12 = 11, College Degree = 25 (Completed = 16, Credits = 9), University Degree = 20 (Completed = 12, Credits = 8), Post-Grad = 5
EMPLOYMENT STATUS	Employed = 33 (Full-Time = 20, Part-Time = 13), Unemployed = 17, Retired = 2, School = 8 (Full-Time = 6, Part-Time 2), Unknown = 1
CLINICAL MEASURES (M ± SD)	
CAPS – TOTAL	41.54 ± 9.39
CTQ – TOTAL	66.52 ± 21.82
CTQ – EMOTIONAL ABUSE	16.40 ± 6.46
CTQ – PHYSICAL ABUSE	10.03 ± 4.55
CTQ – SEXUAL ABUSE	12.27 ± 8.03
CTQ – EMOTIONAL NEGLECT	16.72 ± 5.52
CTQ – PHYSICAL NEGELCT	11.10 ± 4.55
MDI – TOTAL	68.98 ± 29.39
MDI – DISENGAGEMENT	15.53 ± 5.13
MDI – DEPERSONALIZATION	11.70 ± 13.37
MDI – DEREALIZATION	10.80 ± 4.51
MDI – EMOTIONAL CONSTRICTION	13.10 ± 5.66
MDI – MEMORY DISTURBANCE	10.73 ± 4.78
MDI – IDENTITY DISSOCIATION	7.12 ± 4.42

Abbreviations.

PTSD: Post-Traumatic Stress Disorder; CAPS: Clinician-Administered PTSD Scale; CTQ: Childhood Trauma Questionnaire; MDI: Multiscale Dissociation Inventor*y*.

### 2.2 Recording

A 19-channel EEG cap was used to measure whole-scalp activity during a 3-minute, eyes-open baseline recording. Scalp voltages were recorded with an Ag/AgCl electrode cap (Electro-Cap International, Inc. https://www.electro-cap.com) according to the 10–20 international system. The ground electrode was placed on the scalp at a site equidistant between Fpz and Fz. Electrical signals were amplified with the Mitsar 21-channel EEG system (Mitsar-201, CE0537, Mitsar, ltd. https://www.mitsar-medical.com) and all electrodes were kept below 5 kΩ impedance. EEG data were recorded at a sampling rate of 250 Hz using an earlobe-linked referential montage. For export, the files were filtered with a 0.5–130 Hz bandpass filter offline.

### 2.3 EEG preprocessing

The following sequence of steps was performed to remove artifactual (i.e., non-cerebral) sources of electrical activity that may contaminate EEG recordings (Bailey et al., 2022). Firstly, EEG was bandpass filtered at 1–80 Hz. Next, the ZapLine method was used to remove the top 6 components around the 60 Hz main line frequency (de Cheveigné, 2020). Afterward, we removed bad channels using EEGLAB’s *pop_rejchan()* function at a z-score of > 3 and interpolated the rejected channels. Data was then re-referenced to a common average. After, Infomax ICA was performed using the *runica()* function (while estimating and accounting for the reduced rank of the data using PCA). We then rejected specific ICA components related to I.) eye movements using the EyeCatch algorithm default settings (Bigdely-Shamlo et al., 2013) and II.) muscle artifacts flagged by ICLabel at > 50 % probability (Pion-Tonachini et al., 2019). We then automatically removed additional low-frequency artifacts using wavelet ICA at a threshold of 10 and a wavelet level of 10 (Castellanos & Makarov, 2006). Finally, remaining EEG artifacts were removed epoch-wise with a z-score-based method using the FASTER plug-in (Nolan et al., 2010), rejecting 1-second epochs deviating by more than two standard deviations.

### 2.4 Microstate segmentation

#### 2.4.1 Fitting

**Fig. 1:**
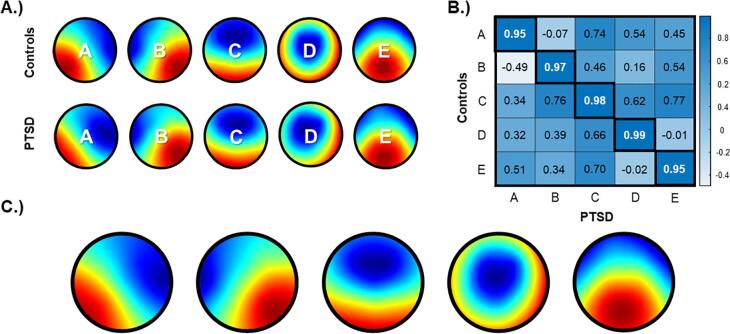
EEG microstate maps. A.) Initially, group-averaged maps were estimated by separately clustering healthy controls (top) and participants with PTSD (bottom). B.) To verify that the group-averaged maps were spatially equivalent, spatial correlations were conducted across all maps, with diagonal correlations representing matching maps (cut-off: r = 0.95). C.) Maps were found to be spatially equivalent group-wise, permitting group-averaged maps to be averaged once more, resulting in a total of five grand mean maps. **(1.5 column, colour).**

De-artifacted datasets were band-passed filtered between 1 and 30 Hz and re-referenced to a common average. Using the microstate plug-in (https://www.thomaskoenig.ch/index/php/software/microstates-in-eeglab) in EEGLAB, EEG microstates were estimated initially at the single-subject level. In particular, a maximum of 1000 global field power (GFP) peaks were selected randomly and submitted to a modified (i.e., polarity-independent) *k*-means clustering with 100 repetitions. *k*-means clustering was performed on cluster numbers ranging from *k* = 4 to *k* = 7. Single-subject maps were then re-ordered by minimizing the average spatial correlation across maps. Next, microstate maps were averaged across all subjects group-wise, resulting in a series of group-averaged maps (*k* = 4 to *k* = 7). We found *k* = 5 to have the highest map reliability, which was estimated as the mean spatial correlation of each subject’s map with the group-averaged map. As shown in Fig. 1B, group-averaged maps (*k* = 5) between PTSD and control groups were spatially correlated to verify that they were approximately equivalent, with a correlation coefficient cut-off of *r* = 0.95. Lastly, PTSD and control group-averaged maps were averaged together, resulting in a final set of five grand mean maps (Fig. 1C).

#### 2.4.2 Backfitting

Next, grand mean maps (*k* = 5) were fitted back to the single-subject data. Backfitting involves assigning all time points to one of the five grand mean maps based on which map displayed the highest spatial correlation to the observed topography at each time point. If the spatial correlation was below *r* = 0.50, the time point was given a non-assigned label. A smoothing window of seven samples (56 ms) was used to ensure temporal continuity of the signals by adjusting the correlation of the central time point with a smoothing factor of 10. Identical label sequences that did not reach a duration of three samples (24 ms) were split into two parts, each sharing the highest spatial correlation with its neighboring segment and relabeled accordingly. Microstate-derived spatiotemporal measures were then computed at the single-subject level and averaged at the group-level, they included:

**Occurrence**: frequency that a given microstate occurs independent of its duration.

**Mean duration**: average duration that a given microstate remains stable.

**Time coverage**: fraction of total time at which a given microstate dominates.

#### 2.4.3 Statistics

Three microstate-derived spatiotemporal measures were compared group-wise with broadband (1–30 Hz), delta (1–4 Hz), theta (4–8 Hz), alpha (8–12 Hz), and beta band (15–30 Hz) microstate maps (A, B, C, D, E) separately using permutation tests of independence on the group means. Since no strong pre-established hypotheses were generated, two-sided tests were used. *p*-values were estimated by simulated random sampling with 10,000 replications. Statistical results were corrected for multiple comparisons using the Bonferroni method (*p*FWE), with 75 independent tests conducted total. Lastly, we used Cohen’s D (*d*) to report effect sizes for each independent test.

### 2.5 Prediction models

#### 2.5.1 Model definition

Linear support vector machine (SVM) classification with ‘L2′ norm penalization and squared hinge loss function was used to classify participants with PTSD using machine learning. Models were generated using five frequency-specific (i.e., delta, theta, alpha, beta, broadband) and five map-specific (i.e., A, B, C, D, E) definitions, with each model including 15 features corresponding to the three microstate-derived spatiotemporal measures calculated within a frequency band and across all microstate maps (i.e., frequency-specific), or within a microstate map and across all frequency bands (i.e., map-specific). Hence, the number of features in each model was held constant, allowing the models to be compared without biasing the dimensionality of one in favour of another. Lastly, all models were fitted with z-scored standardized features, removing each respective mean and scaling them to unit variance.

#### 2.5.2 Model evaluation

Before comparing the models, a specific number of top features has to be selected (e.g., *k =* 3). Reducing the number of features helps to avoid model overfit and increase model performance (by avoiding the so-called ‘curse of dimensionality’). In MATLAB, we initially explored three different algorithms for reducing the feature set, they included I.) Neighbourhood Component Analysis (NCA; function *fscnca ()*), II.) Minimum Redundancy – Maximum Relevance (MRMR; function *fscmrmr ()*) and III.) ReliefF. Each of these algorithms function to rank the features based on their individual prediction score. To determine which algorithm to ultimately use, we compared all three algorithms across the total feature space over 10 runs (*k* = 75, *k*-folds = 10). Models were compared using mean accuracy and the area under the curve (AUC). Based on these measures, we found that ReliefF performed the best overall (see Supplemental Materials). Hence, in the analyses to follow, ReliefF with *k*-nearest neighbours = 15 was used to reduce the corresponding feature spaces (*k* = 1:15). Since all the models started with the same number of features (i.e., 15), we held the number of nearest neighbours constant throughout the analyses.

10 times repeated 10-fold cross-validation tests were conducted to estimate and compare models (i.e., 10 × 10; Bouckaert, 2003). In each run, nine-folds (i.e., 90 %) of the sampled subjects were used to train the model, while one-fold (i.e., 10 %) of the sampled subjects were used to evaluate the model. To avoid selecting a specific number of features, we compared the models across the full range of features based on the 10 × 10 mean accuracy (*k* = 1:15). Moreover, direct comparisons were conducted at *k* = 1 and *k* = 15 (since the former was generally shown to have the highest mean accuracy and the latter included all the features). Models were evaluated using several diagnostic measures:

**Accuracy:** number of samples predicted correctly out of the testing set.

**Sensitivity**: index of a model’s ability to predict true positives (i.e., true positive rate).

**Specificity**: index of a model’s ability to predict true negatives (i.e., false positive rate).

**AUC**: the area under the ROC curve, a metric that aggregates across all the possible discrimination thresholds to give an overall measure of model performance indexed between 0 (a model with a 100 % error rate, i.e., no correct predictions) and 1 (a model with a 0 % error rate, i.e., all correct predictions).

### 2.6 Correlations with clinical measures

A single model was trained using all available broadband and frequency-specific microstate measures, combining the 15 features included in each of the five frequency-specific models. The aggregated, 75 feature space was reduced to the top ranked feature. The top ranked feature was then used to conduct a clinical correlation with PTSD symptom severity scores (CAPS; Blake et al., 1995, Weathers et al., 2018) using a two-sided permutation test (1000 permutations) on the Pearson’s correlation coefficients. The top ranked feature was selected to reduce the number of clinical correlations, focusing only on the feature found to be the most discriminant. A clinical correlation with PTSD symptom severity was only conducted across participants with PTSD (N = 61), since healthy controls were not administered the CAPS-IV or CAPS-5. Of note, Ros and colleagues (2017) assessed PTSD symptom severity based on CAPS-IV diagnostic criteria, while Nicholson and colleagues (2020) assessed PTSD symptoms with the CAPS-5. These total symptom scores were normalized, permitting clinical correlations to be conducted. However, since the CAPS-IV includes additional subscales not included in the CAPS-5, we did not explore clinical correlations among CAPS subscales.

## 3 Results

### 3.1 Microstate configurations

Microstates were segmented independently for each group (i.e., PTSD, healthy controls) to identify any differences in the maps group-wise. Group-averaged maps were found to be spatially equivalent, with the two groups displaying spatial correlations between maps exceeding 95 % topographic similarity (i.e., *r* > 0.95) (Fig. 1B). As can be seen in Fig. 1A, both groups produced the typical (canonical) microstate maps reported in the literature, namely maps with diagonal orientations (A and B), an anterior-posterior orientation (C), a fronto-central maximum (D), and a centro-posterior maximum (E).

### 3.2 Microstate-derived spatiotemporal measures

#### 3.2.1 Occurrence

**Fig. 2:**
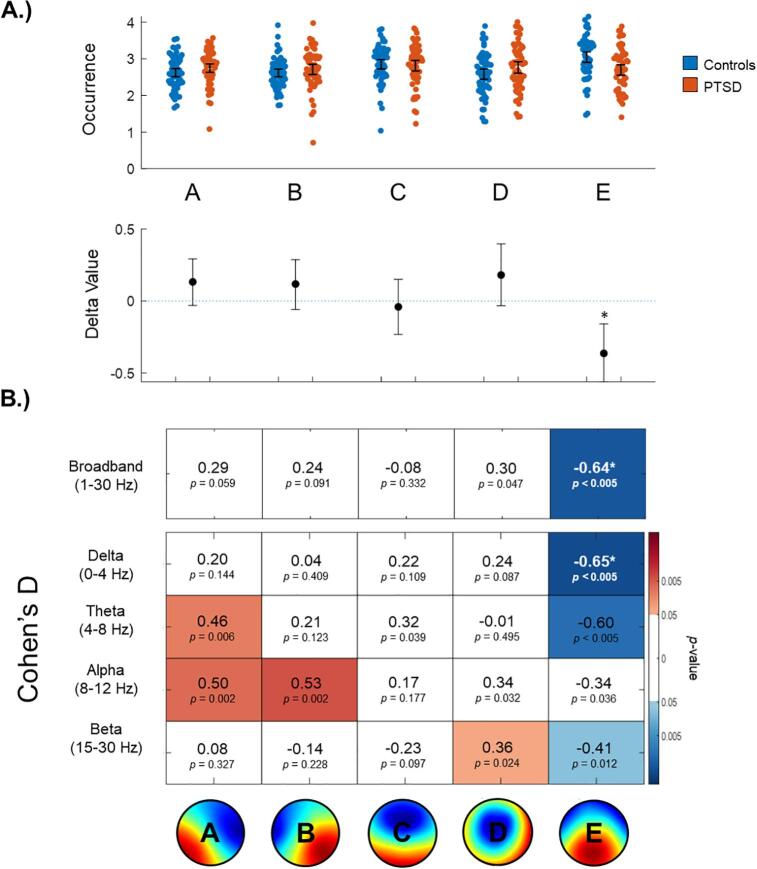
Occurrence of broadband and frequency-specific microstates. A.) On the top, broadband occurrence values are plotted using single-subject data. On the bottom, ‘delta values’ indicate absolute differences between the group means with 95 % confidence intervals. Asterisks denote significance at a corrected threshold of pFWE < 0.05. Identical plots specific to each frequency band are provided in the Supplemental Materials. B.) Each box includes a Cohen’s D effect size between participants with PTSD and controls, and an uncorrected p-value corresponding to the permutation test on the group means. A red and a blue box indicates a positive or a negative difference, respectively (i.e., p(uncorrected) < 0.05), with darker shades indicating a greater difference. Asterisks denote significance at a corrected threshold of pFWE < 0.05. **(2 column, colour).** (For interpretation of the references to colour in this figure legend, the reader is referred to the web version of this article.)

Statistically significant (FWE-corrected) results were limited to microstate map E. As depicted in Fig. 2, broadband microstate map E (*d* = -0.64, *p*FWE = 0.03) and delta band microstate map E (*d* = -0.65, *p*FWE = 0.02) were found to occur significantly less frequently in participants with PTSD as compared to controls.

#### 3.2.2 Mean duration

**Fig. 3:**
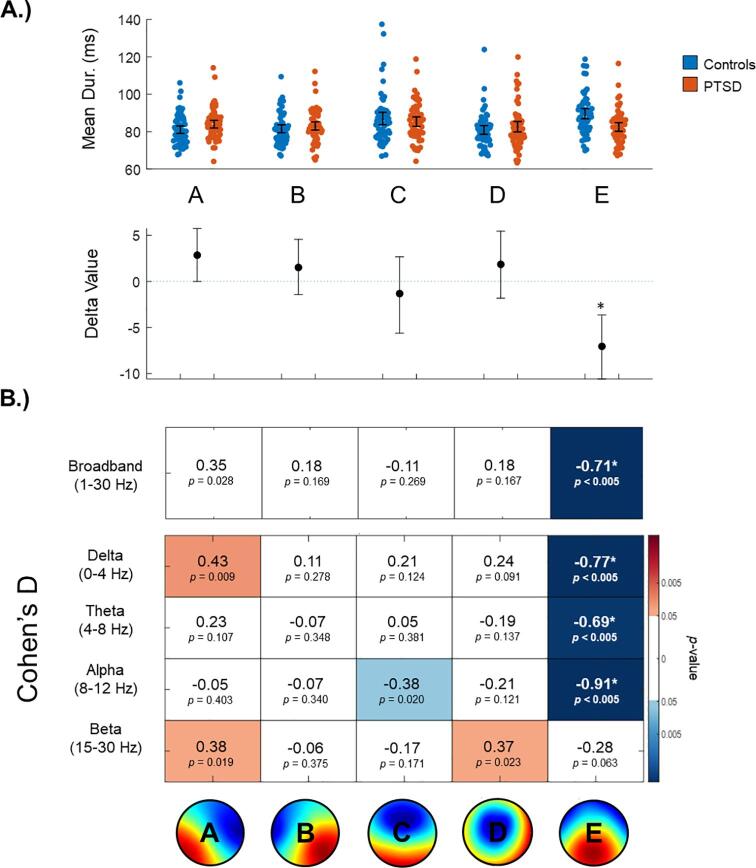
Mean duration of broadband and frequency-specific microstates. A.) On the top, broadband mean duration values are plotted using single-subject data. On the bottom, ‘delta values’ indicate absolute differences between the group means with 95 % confidence intervals. Asterisks denote significance at a corrected threshold of pFWE < 0.05. Identical plots specific to each frequency band are provided in the Supplemental Materials. B.) Each box includes a Cohen’s D effect size between participants with PTSD and controls, and an uncorrected p-value corresponding to the permutation test on the group means. A red and a blue box indicates a positive or a negative difference, respectively (i.e., p(uncorrected) < 0.05), with darker shades indicating a greater difference. Asterisks denote significance at a corrected threshold of pFWE < 0.05. **(2 column, colour)**. (For interpretation of the references to colour in this figure legend, the reader is referred to the web version of this article.)

Statistically significant (FWE-corrected) results were limited to microstate map E. As illustrated in Fig. 3, broadband (*d* = -0.71, *p*FWE < 0.01), delta band (*d* = -0.77, *p*FWE < 0.01), theta band (*d* = -0.69, *p*FWE < 0.01), and alpha band microstate maps E (*d* = -0.91, *p*FWE < 0.01) were all found to have a significantly shorter mean duration in participants with PTSD as compared to controls.

#### 3.2.3 Time coverage

**Fig. 4:**
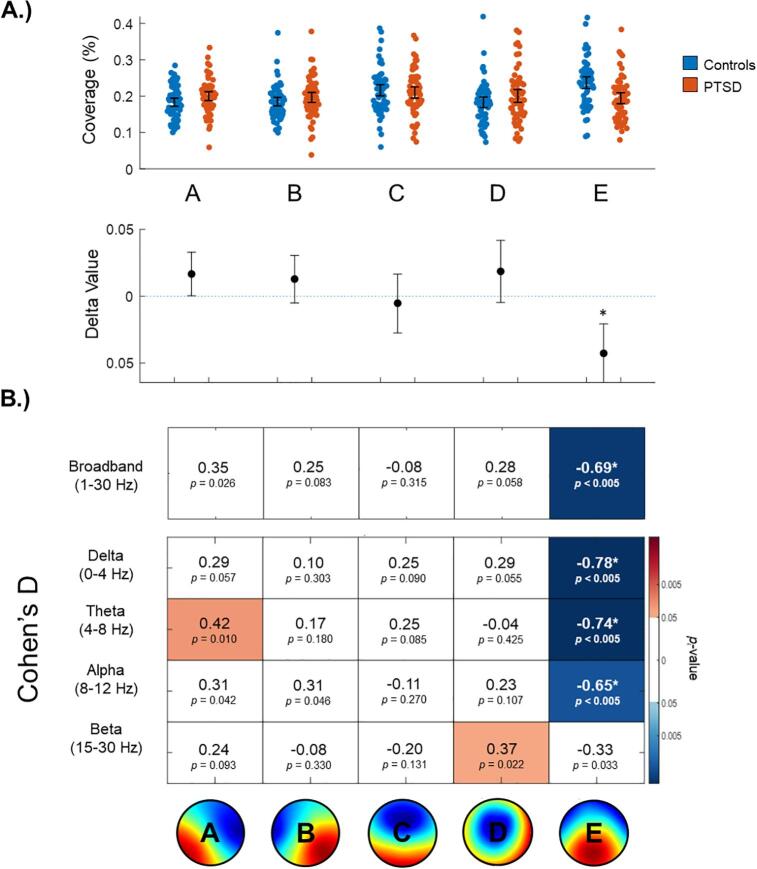
Time coverage of broadband and frequency-specific microstates. A.) On the top, broadband time coverage values are plotted using single-subject data. On the bottom, ‘delta values’ indicate absolute differences between the group means with 95 % confidence intervals. Asterisks denote significance at a corrected threshold of pFWE < 0.05. Identical plots specific to each frequency band are provided in the Supplemental Materials. B.) Each box includes a Cohen’s D effect size between participants with PTSD and controls, and an uncorrected p-value corresponding to the permutation test on the group means. A red and a blue box indicates a positive or a negative difference, respectively (i.e., p(uncorrected) < 0.05), with darker shades indicating a greater difference. Asterisks denote significance at a corrected threshold of pFWE < 0.05. **(2 column, colour)**. (For interpretation of the references to colour in this figure legend, the reader is referred to the web version of this article.)

Once again, statistically significant (FWE-corrected) results were limited to microstate map E. As shown in Fig. 4, broadband (*d* = -0.69, *p*FWE = 0.03), delta band (*d* = -0.78, *p*FWE < 0.01), theta band (*d* = -0.74, *p*FWE = 0.01), and alpha band microstate maps E (*d* = -0.65, *p*FWE = 0.02) were all found to have a significantly reduced time coverage in participants with PTSD as compared to controls, an expected result when considering that microstate map E was found to occur less frequently and have a shorter mean duration in participants with PTSD.

### 3.3 Classification of PTSD vs healthy controls using machine learning

In the following sections, we trained SVM models to predict the class membership of an overall sample of participants with PTSD (N = 61) and healthy controls (N = 61) using 10-fold cross-validation (see Methods for more details). In the Supplemental Materials, classification accuracies of every frequency-specific SVM model was compared to a corresponding null (surrogate) model. All accuracies were averaged over ten runs. Across all the possible *k*-values (i.e., numbers of features), frequency-specific models significantly out-performed the corresponding null models, demonstrating that randomly permutating group membership of participants does not produce measurable learning.

#### 3.3.1 Frequency-specific models

**Fig. 5:**
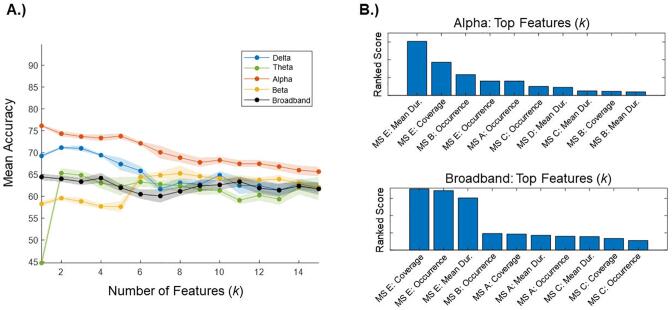
Frequency-specific models. A.) The mean accuracy of five frequency-specific models were compared. Mean accuracy values are provided across the full range of possible features, with the alpha band model consistently having the highest classification accuracy. Shaded areas represent 95% confidence intervals. B.) Using ReliefF, we ranked the top 10 features of the alpha band model as compared to the broadband model. The higher the ranked score, the more strongly the feature contributed to the classification task. **(1.5 column, colour)**.

Five frequency-specific models (delta, theta, alpha, beta, and broadband) were compared across all the possible numbers of features (*k*-values = 1:15). Over 10 runs, the alpha band model out-performed all the other models (Fig. 5A), with the top feature alone (i.e., *k* = 1) revealing the highest classification accuracy. In the alpha band model, the top feature corresponded to the *mean duration of microstate map E*, while the *time coverage of microstate map E* ranked highest with respect to the broadband model (Fig. 5B).

At *k* = 1, the top feature of the alpha band model out-performed the top feature of the broadband model (mean accuracy: alpha = 76 %, broadband = 65 %). In terms of classification sensitivity and specificity, the alpha band model out-performed the broadband model (sensitivity: alpha = 79 %, broadband = 72 %; specificity: alpha = 74 %, broadband = 62 %). Statistically, the alpha band model significantly out-performed the broadband model in terms of the AUC (alpha = 0.75, broadband = 0.71, *p* = 0.03).

At *k* = 15, the full alpha band model performed similarly to the full broadband model (mean accuracy: alpha = 66 %, broadband = 62 %). The alpha band model had a higher classification sensitivity (alpha = 82 %, broadband = 75 %), while the broadband model had a higher classification specificity (alpha = 66 %, broadband = 72 %). Statistically, the full alpha band model and the full broadband model did not differ significantly in terms of the AUC (alpha = 0.69, broadband = 0.66, *p* = 0.22).

#### 3.3.2 Map-specific models

**Fig. 6:**
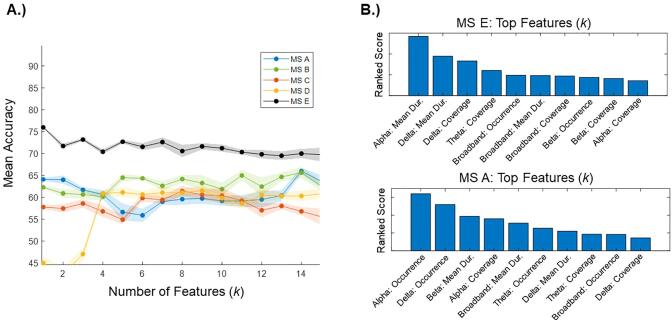
Map-specific models. A.) The mean accuracy of five map-specific models were compared. Mean accuracy values are provided across the full range of possible features, with the microstate map E model consistently having the highest classification accuracy. Shaded areas represent 95% confidence intervals. B.) Using ReliefF, we ranked the top 10 features of the first and the second most accurate models. The higher the ranked score, the more strongly the feature contributed to the classification task. **(1.5 column, colour)**.

With respect to the five map-specific models, the microstate map E model out-performed all the other models (Fig. 6A), with the top feature alone (i.e., *k* = 1) revealing the highest classification accuracy. In the microstate map E model, the top feature corresponded to the *mean duration of the alpha band map* (same feature as the alpha band model). In the microstate map A model (second best), the top feature corresponded to the *occurrence of the alpha band map* (Fig. 6B).

At *k* = 1, the top feature of the microstate map E model out-performed the top feature of the microstate map A model (mean accuracy: map E = 76 %, map A = 65 %). Similarly, concerning classification sensitivity and specificity, the microstate map E model out-performed the microstate map A model (sensitivity: map E = 79 %, map A = 62 %; specificity: map E = 74 %, map A = 57 %). Statistically, the microstate map E model significantly out-performed the microstate map A model in the AUC (map E = 0.75, map A = 0.64, *p* = 0.01).

At *k* = 15, the full microstate map E model out-performed the full microstate map A model (mean accuracy: map E = 70 %, map A = 64 %), revealing a higher classification sensitivity (map E = 72 %, map A = 72 %) and specificity (map E = 80 %, map A = 72 %). Statistically, the microstate map E model and the microstate map A model did not differ significantly in the AUC (map E = 0.74, map A = 0.70, *p* = 0.14).

### 3.4 Feature-specific classification accuracies

**Fig. 7:**
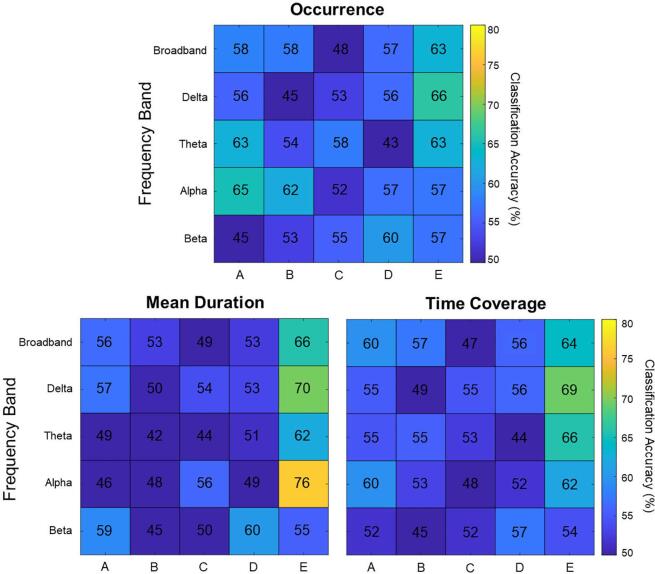
**Heat plots demonstrating classification accuracy of all features.** Individual classification accuracies of every microstate-based feature have been provided. Warmer colours indicate a stronger classification accuracy, with the mean duration of alpha band microstate map E found to be the best individual predictor of PTSD diagnosis. **(2 column, colour)**.

In addition to ranking the features using ReliefF, we also calculated the mean classification accuracy of every feature independent of the SVM models. As illustrated in Fig. 7, only microstate map E features had reasonably high classification accuracies, with *the mean duration of alpha band microstate map E* having the highest individual classification accuracy at 76 %.

### 3.5 Correlations with clinical measures

A single aggregate model was trained using all available microstate measures. The top ranked feature was selected to conduct a clinical correlation with PTSD symptom severity. This correlation was not found to be significant at a corrected threshold of *p*FWE < 0.05.

## 4 Discussion

### 4.1 Overview

In the present study, we sought to examine fast-scale brain dynamics in participants with PTSD using a microstate framework. Significant group differences were observed solely in the centro-posterior maximum microstate map E, with large effect sizes. In the broadband comparisons, microstate map E occurred significantly less frequently and had a significantly shorter mean duration in participants with PTSD as compared to controls. These differences were reflected in the narrow frequency bands as well, with lower frequency bands like delta, theta, and alpha repeating these broadband differences, only with larger effect sizes. These findings were corroborated by a machine learning classification analysis, which found that a model containing only alpha band features significantly out-performs a model containing broadband features at classifying PTSD. To the best of our knowledge, these results constitute the first evidence of EEG microstate abnormalities in PTSD, whilst demonstrating that filtering EEG into distinct frequency bands improves microstate-based classification of a psychiatric disorder. Hence, frequency-specific EEG microstates may offer a way to simultaneously capture the spectral and the temporal features of resting-state EEG.

### 4.2 Microstate map E and alpha band rhythms

Our main finding is that microstate map E was temporally under-represented in PTSD: evidenced by significantly lower occurrence, mean duration, and time coverage relative to controls. In the context of brain state dynamics, this would be indicative of less stability and reduced dwell time of the cortical generator(s) of map E, coinciding with ‘shallower’ attractor basins.

Early work by Lehmann and colleagues (1987, 2005), Koenig and colleagues (1999), and others (Britz et al., 2010, Brodbeck et al., 2012, Khanna et al., 2014, Milz et al., 2016) did not define a fifth microstate map, opting for a four-cluster solution (*k* = 4) instead. Custo et al. (2017), on the other hand, used a seven-cluster solution (*k* = 7) and produced a map with a configuration closely resembling microstate map E, with sources estimated in the dorsal anterior cingulate cortex, the inferior frontal gyrus, and the insula. These regions have been implicated in the salience network, a network mediating bottom-up attention processing (Uddin, 2015). Interestingly, the salience network has been found to be dysregulated in PTSD (Akiki et al., 2017, Abdallah et al., 2019, Nicholson et al., 2020), which has been suggested to be mediating attention-related biases toward threat (Sripada et al., 2012), as well as hypervigilance symptomatology broadly (Patel et al., 2012, Koch et al., 2016).

When investigating visual cortical activity, Clancy and colleagues (2017) found significantly reduced alpha band activity in participants with PTSD as compared to participants with general anxiety disorder and healthy controls, suggesting that these differences were specific to PTSD, not anxiety disorders more generally. Consistent with parallel work by Ros and colleagues (2017), reduced alpha band activity in participants with PTSD was proposed to be a hallmark of sensory disinhibition, a phenomenon defined by reduced cortical inhibition and enhanced cortical hyper-excitability. Elsewhere, reduced alpha oscillations have been found to be directly associated with states of cortical hyper-excitability (Romei et al., 2008), with low-level sensory signals being promoted to consciousness more easily (e.g., phosphenes). Under normal conditions, only those sensory signals deemed to be salient would be delivered to conscious processing, a process thought to be coordinated by the salience network and alpha oscillations (Sadaghiani et al., 2010). Therefore, it may be the case that microstate map E, and, in particular, its alpha band-driven cortical disinhibition, could be contributing to hypervigilance symptoms in participants with PTSD, which would help explain why it demonstrated the strongest microstate-based differences.

Importantly, not all researchers agree that microstate map E represents the electrophysiological fingerprint of the salience network. In fact, Britz and colleagues found instead that microstate map C was correlated with activity in salience network-related brain regions, although, as discussed, they did not include a microstate matching that of microstate map E. Moreover, correlation coefficients between Britz and colleagues’ EEG-informed model of microstate map C activity and the salience network were relatively modest (i.e., *r* = 0.44; Britz et al., 2010). Nonetheless, the initial labelling provided by Britz and colleagues has persisted, with multiple studies referencing microstate map C as being mediated by salience network-related activity (Nishida et al., 2013, Khanna et al., 2015, Hu et al., 2021). In common, microstate map C and microstate map E both have topographic orientations with polarities along the anterior-posterior axis, suggesting that, perhaps, these maps have common oscillatory generators – a research question worth future inquiry.

### 4.3 Classification of PTSD using frequency-specific microstates

When Lehmann (1987) first introduced microstate-based segmentation, he did so based on alpha band EEG, as opposed to broadband EEG. However, the majority of microstate-based analyses to date have exclusively segmented broadband EEG (Michel & Koenig, 2018). Férat and colleagues (2022) recently applied spectral filtering prior to microstate-based segmentation, finding frequency-specific microstate maps to be spatially equivalent to the broadband maps, yet differing in terms of their temporal dynamics. In healthy participants, Férat and colleagues found that alpha band microstates were more accurate predictors of eyes-open vs eyes-closed conditions relative to broadband measures. We arrive at a similar conclusion, revealing a model containing alpha band features to significantly out-perform a model containing broadband features. Different frequency bands relate to different oscillatory generators, with low-frequency activity found to be produced more so by thalamic and limbic generators serving global synchrony, while high-frequency activity has been found to be produced more so by higher-order, cortical regions (Ganzetti & Mantini, 2013; although see Groppe et al., 2013, Keitel et al., 2016). Hence, frequency-specific models might yield a much richer description of pathological EEG dynamics, improving microstate-based classification and opening up the possibility to discover more specific neuromarkers underlying a variety of psychiatric disorders.

### 4.4 Limitations and future directions

We offer a few limitations for consideration. Firstly, around half of the participants with PTSD were currently prescribed psychotropic medication, adding a potential confound to the data. However, patients with PTSD are prescribed medication commonly, and hence any effort to control for these effects would have reduced the generalizability of the findings to the population at large. Secondly, we used a 19-channel cap to collect EEG, favoring a more affordable and accessible clinical device instead of one with a higher density electrode array. However, unlike source-space EEG analyses, EEG microstates do not appear to be compromised at lower channel densities (Khanna et al., 2014), a key benefit associated with these analyses. Thirdly, EEG analyses have a common limitation known as the inverse-source problem. In short, EEG records electric potential field patterns over the scalp (i.e., surface maps); however, different patterns of source activation (i.e., generators) can produce the same surface map. Hence, we cannot say confidently which regions are underlying these EEG microstates. Fourthly, although we had the largest clinical sample of participants with PTSD used in a microstate analysis to date, it was not large enough to permit creating a hold-out test set when performing machine learning. Creating a hold-out test set with our current sample size would have significantly reduced the amount of training data, potentially leaving too few subjects to train a robust model. More broadly, smaller sample sizes – like that represented here – have been found to inflate classification accuracies (Varoquaux, 2018), with smaller datasets sometimes yielding higher accuracies than larger ones (Flint et al., 2021). With this in mind, we encourage future work to look to replicate these findings with larger datasets. In doing so, we might find that these accuracies are slightly inflated, although we would hypothesize that the relative differences in feature-specific accuracies would persist.

## 5 Conclusion

In the present study, we investigated fast-scale brain dynamics using an EEG microstate framework, revealing robust differences in participants with PTSD. Microstate-based segmentation allowed us to isolate these differences to specific spatial configurations, with microstate map E (with centro-posterior maximum) demonstrating significantly less presence in PTSD. Given that narrowband microstate models obtained the highest classification accuracies of around 75 %, our analyses support frequency-specific microstate segmentation as a valuable adjunct to broadband segmentation when classifying psychiatric disorders. If replicated, these results suggest EEG microstates may serve as promising clinical neuromarkers of PTSD – helping not only to identify PTSD, but to inform neuromodulatory therapies like microstate-based neurofeedback (Hernandez et al., 2016). If it turns out that microstate map E represents a reliable electrophysiological fingerprint of the salience network, then perhaps targeting microstate map E could serve as an effective way of addressing salience network-related overactivity in PTSD, offering a cost-effective alternative to real-time fMRI neurofeedback in patient populations.

## CRediT authorship contribution statement

**Braeden A. Terpou:** Conceptualization, Formal analysis, Visualization, Writing – original draft, Writing – review & editing. **Saurabh B. Shaw:** Writing – review & editing. **Jean Théberge:** Funding acquisition, Investigation, Resources. **Victor Férat:** Methodology, Software. **Christoph M. Michel:** Funding acquisition, Resources, Supervision. **Margaret C. McKinnon:** Funding acquisition, Supervision. **Ruth A. Lanius:** Data curation, Funding acquisition, Investigation, Supervision, Writing – review & editing. **Tomas Ros:** Conceptualization, Data curation, Investigation, Methodology, Resources, Software, Supervision, Writing – review & editing.

## Declaration of Competing Interest

The authors declare that they have no known competing financial interests or personal relationships that could have appeared to influence the work reported in this paper.
