# Supplementary material for: Spectral decomposition of EEG microstates in post-traumatic stress disorder
Source: Neuroimage Clin. 2022 Jul 29;35:103135. doi: 10.1016/j.nicl.2022.103135 (PMC9421541; doi:10.1016/j.nicl.2022.103135)
Supplement: Supplementary data 1 [file mmc1.docx]

**Supplemental Materials**

*spectral decomposition of eeg microstates in post-traumatic stress disorder*

Braeden A. Terpou^1,6,*^, Saurabh B. Shaw^3,6,7^, Jean Théberge^3,4,5^, Victor Férat^8^, Christoph M. Michel^8,9^, Margaret C. McKinnon^1,2,6^, Ruth A. Lanius^3,5,6,†^, & Tomas Ros^8,9,†^

*^1^Department of Psychiatry and Behavioural Neurosciences, McMaster University, Hamilton, Canada;*

*^2^Mood Disorders Program, St. Joseph’s Healthcare Hamilton, Hamilton, Canada;*

*^3^Department of Psychiatry, Western University, London, Canada;*

*^4^Department of Medical Biophysics, Western University, London, Canada;*

*^5^Imaging Division, Lawson Health Research Institute, London, Canada;*

*^6^Homewood Research Institute, Guelph, Canada;*

*^7^Vector Institute, Toronto, Canada;*

*^8^Department of Basic Neurosciences, University of Geneva, Geneva, Switzerland;*

*^9^Centre for Biomedical Imaging (CIBM), Lausanne-Geneva, Switzerland*

**S.1 Feature selection algorithms**

In MATLAB, three different feature selection algorithms were administered to rank the features based on their prediction score, with the hopes of reducing the total number of selected features (*k*) when comparing models (to avoid the so-called ‘curse of dimensionality’). As shown in Figure S1, neighbourhood component analysis (NCA), minimum redundancy – maximum relevance (MRMR), and ReliefF algorithms performed similarly at lower *k*-values, likely since all the algorithms identified the same couple of features as being most discriminant. However, at higher *k*-values, ReliefF (in orange) had an edge over the other algorithms, both with respect to the mean accuracy (runs = 10, *k*-folds = 10), as well as the area under the curve (AUC). Hence, we selected ReliefF as the feature selection algorithm in the analyses.


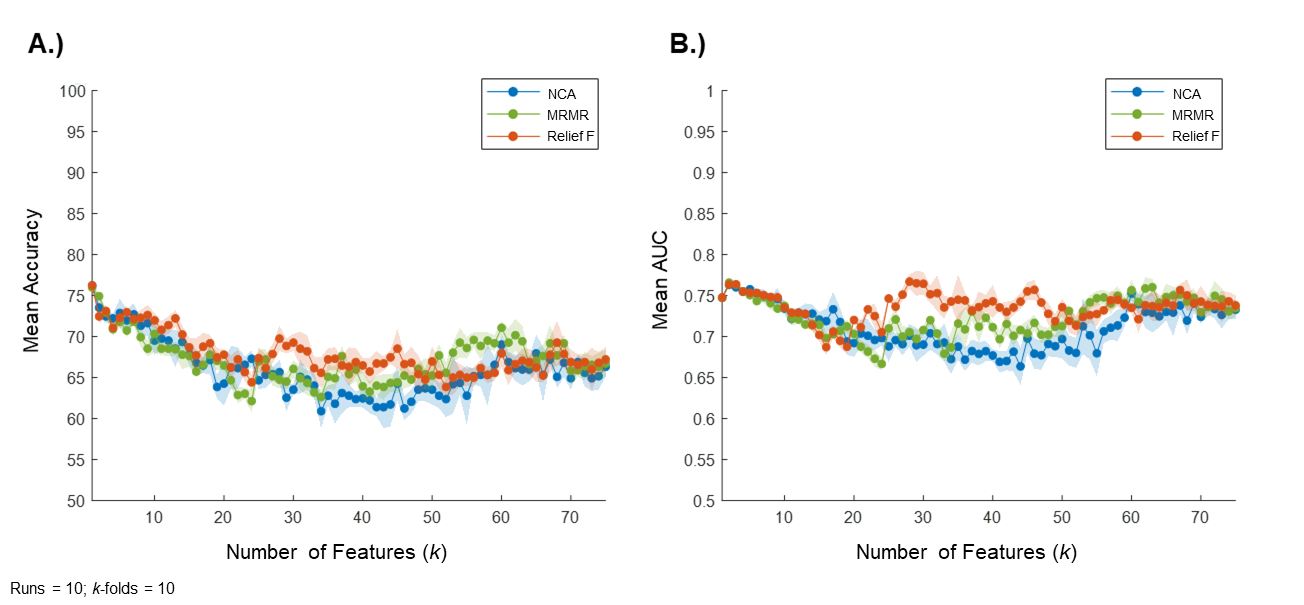


**Figure S1: Feature selection algorithms**

*Three different feature selection algorithms (NCA: blue; MRMR: green; ReliefF: orange) were compared with respect to their mean accuracy (A) and the area under the curve (B) at predicting participant diagnosis across all the possible number of features (k-values). Between k = 25 to k = 35, ReliefF out-competes NCA and MRMR, with shaded areas representing 95% confidence intervals.*

**S.2 Frequency-specific group estimation plots**

In the main manuscript, group estimation plots were only provided for broadband comparisons. Here, we included all the group estimation plots for data visualization purposes.

***
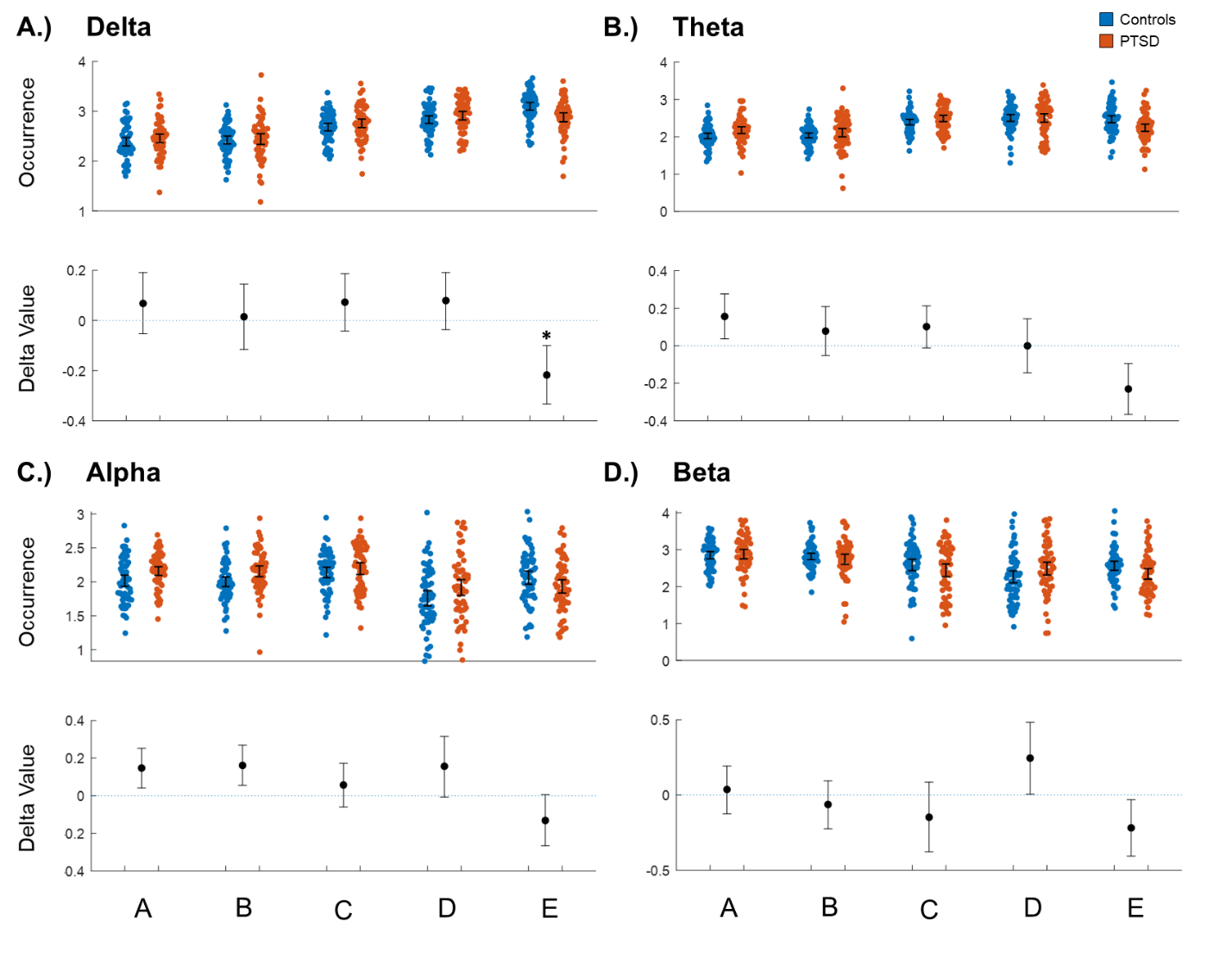
Figure S2: Frequency-specific (spectral) microstate map occurrences***

*Group comparisons were conducted across all microstate-derived spatiotemporal measures. Occurrence differences based on the group means are provided above, with delta band (A), theta band (B), alpha band (C), and beta band (D) occurrence values, variances, and delta values plotted for each microstate and each frequency band combination. Asterisks denote significance at a correction threshold of pFWE < 0.05.*


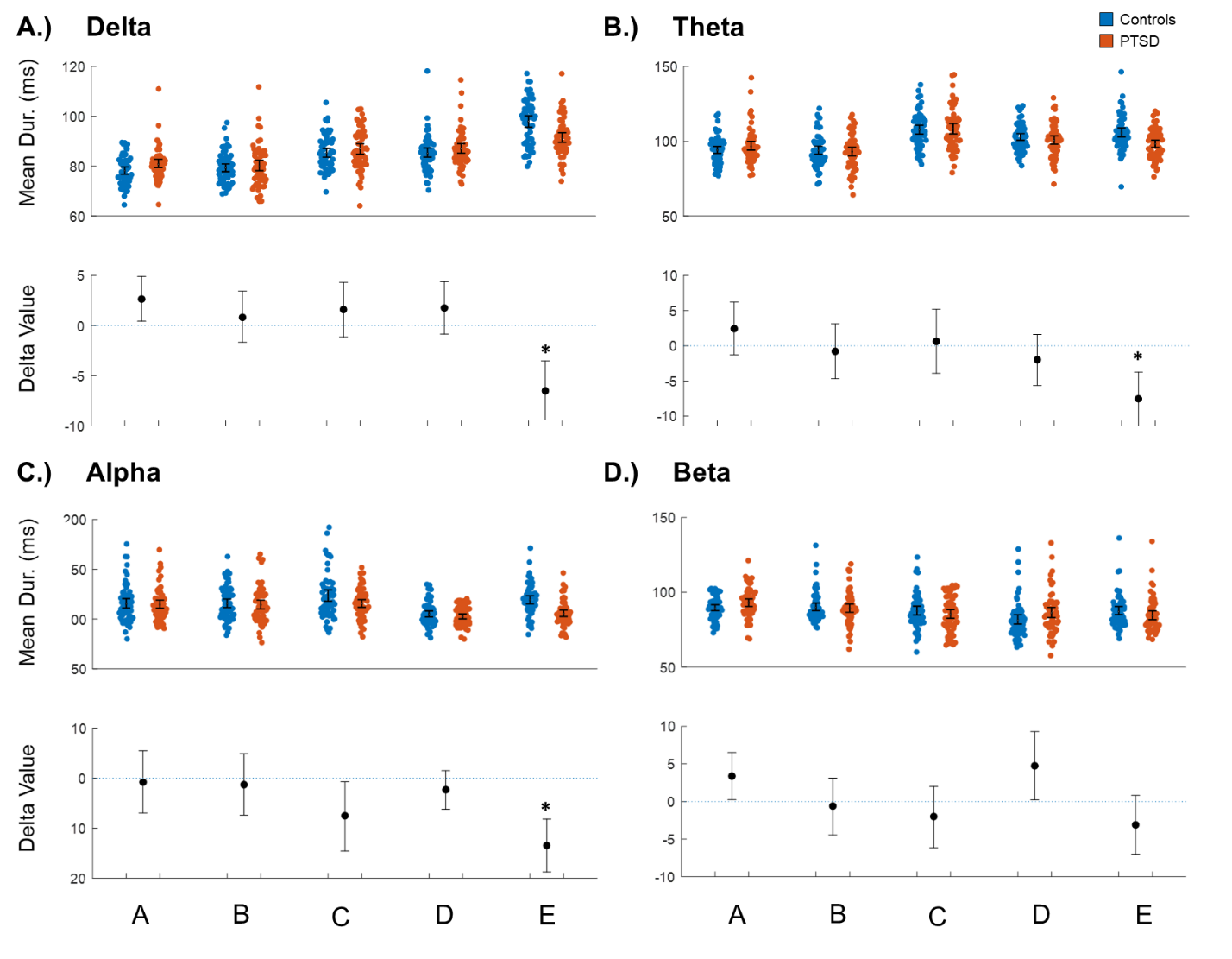


***Figure S3: Frequency-specific (spectral) microstate map mean durations***

*Group comparisons were conducted across all microstate-derived spatiotemporal measures. Mean duration (ms) differences based on the group means are provided above, with delta band (A), theta band (B), alpha band (C), and beta band (D) mean durations, variances, and delta values plotted for each microstate and each frequency band combination. Asterisks denote significance at a correction threshold of pFWE < 0.05.*


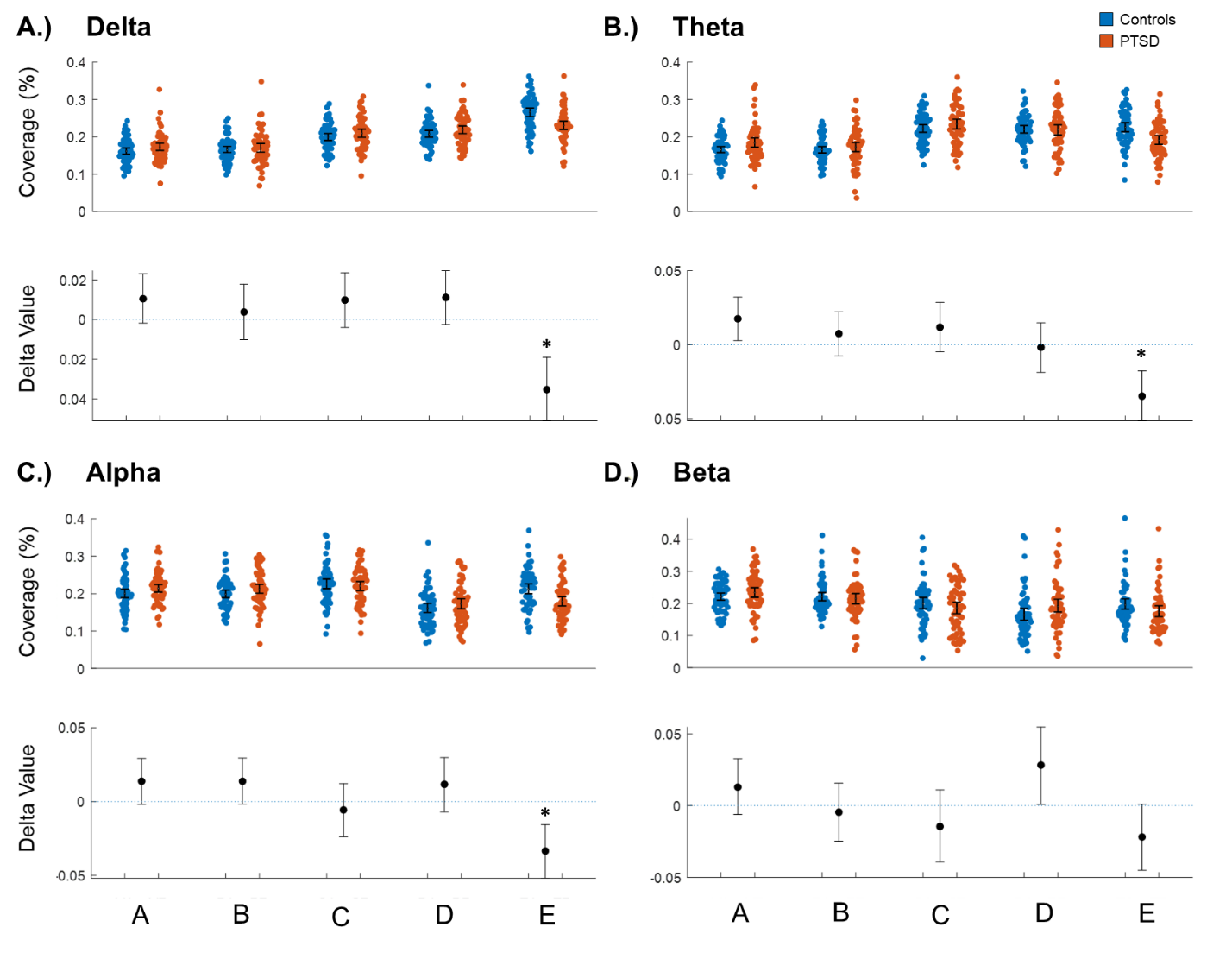


***Figure S4: Frequency-specific (spectral) microstate map coverages***

*Group comparisons were conducted across all microstate-derived spatiotemporal measures. Coverage (%) differences based on the group means are provided above, with delta band (A), theta band (B), alpha band (C), and beta band (D) coverages, variances, and delta values plotted for each microstate and each frequency band combination. Asterisks denote significance at a correction threshold of pFWE < 0.05.*

**S.3 Support vector machine (SVM) model comparisons**

In the main manuscript, we compared models with respect to their 10 x 10 mean accuracy over ten runs (*k*-folds = 10). Here, we offer two more metrics to compare models, namely the balanced accuracy and the AUC. Similar to Figure 5 and Figure 6 in the manuscript, we compared the mean balanced accuracy and the mean AUC across the five frequency-specific (Figure S5) and the five map-specific models (Figure S6). Of note, the same trends hold true, namely that the alpha band model wins in the frequency-specific comparisons, while the microstate map E model wins in the map-specific comparisons. Interestingly, the alpha band model declines slowly in its mean balanced accuracy and mean AUC with increasing numbers of features (k), while the microstate map E model stays relatively consistent with varying *k*-values, demonstrating an appreciable advantage over the other microstate models. Hence, the alpha band and the microstate map E in conjunction seem to perform best at classifying PTSD using resting-state EEG.

**S.3.1 Frequency-specific models**

**
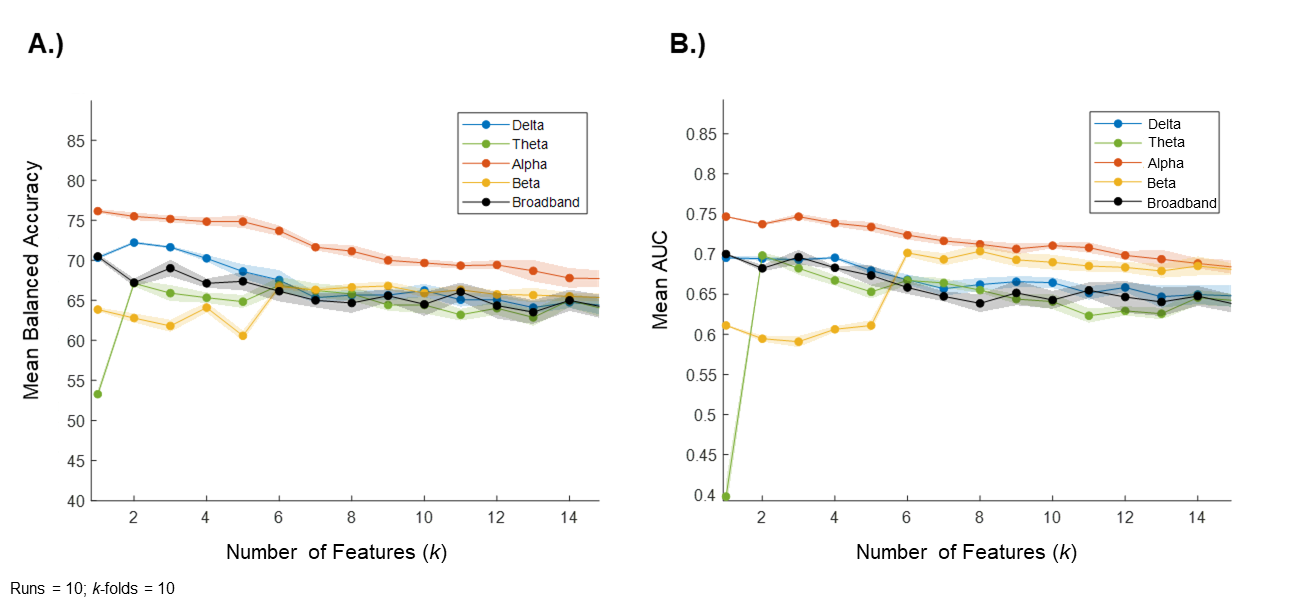
**

***Figure S5: Frequency-specific model comparisons***

*In addition to mean 10 x 10 accuracy, frequency-specific SVM models were compared with respect to mean balanced accuracy and AUC. Similar to the trends for the 10 x 10 mean accuracy, the alpha band model consistently out-performed the other models. Shaded areas represent 95% confidence intervals.*

**S.3.2 Map-specific models**

**
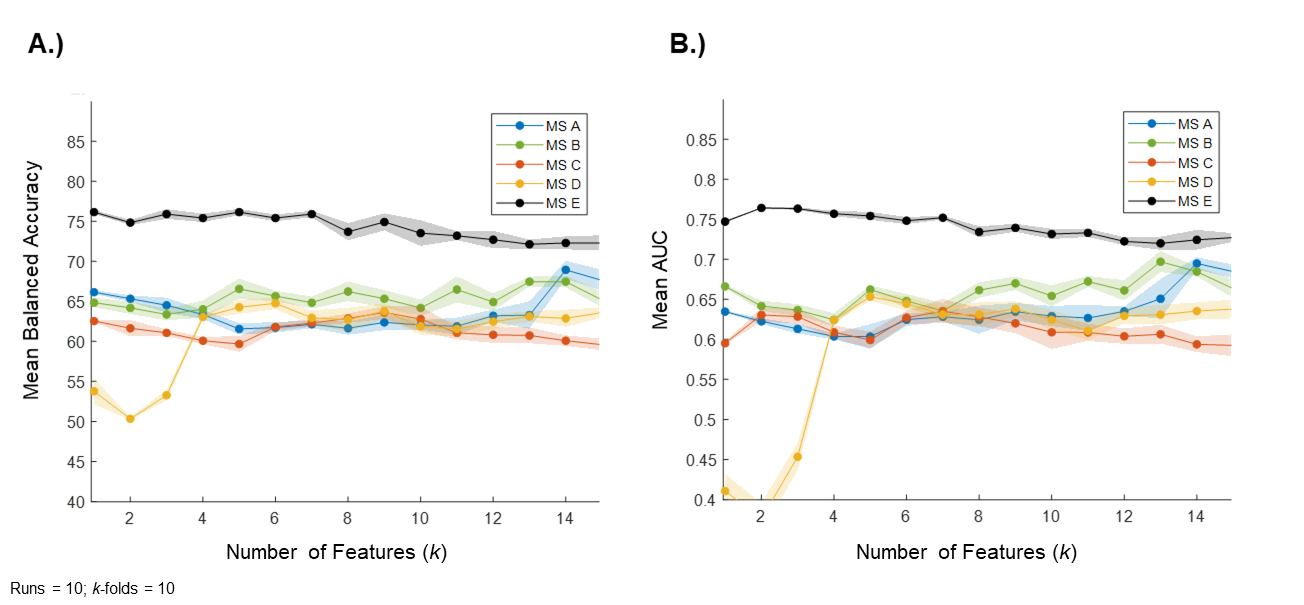
**

***Figure S6: Map-specific model comparisons***

*In addition to mean 10 x 10 accuracy, map-specific models were compared with respect to mean balanced accuracy and AUC. Similar to the trends for the 10 x 10 mean accuracy, the microstate map E model consistently out-performs the other models. Shaded areas represent 95% confidence intervals.*

**S.3.3 Null (Surrogate) Model Comparisons**

For completeness, all balanced and 10 x 10 cross-validation accuracies for the frequency-specific models were compared to a null model, with both sets of accuracies averaged over ten runs (Table S1). Null models included all the same model features as the frequency-specific models; however, PTSD and healthy control labels were randomly permutated for the training set, which constituted 30% of the data. Hence, by comparing models to that of a null model, it allowed us to determine whether models trained using standard machine learning procedures out-perform models trained using misidentified data. In every comparison, balanced and 10 x 10 cross-validation accuracies for the frequency-specific models were found to be significantly higher than that of the null models.

**Table S1: Null (surrogate) SVM model comparisons**

| *k* | Spectral Bands | Balanced Accuracy | 10 x 10 Accuracy | Null Accuracy | *k* | Spectral Bands | Balanced Accuracy | 10 x 10 Accuracy | Null Accuracy |
| --- | --- | --- | --- | --- | --- | --- | --- | --- | --- |
| 1 | Delta | 70.74*** | 69.59*** | 49.19 | 2 | Delta | 71.48*** | 70.66*** | 48.28 |
|  | Theta | 54.43*** | 43.77* | 48.79 |  | Theta | 67.50*** | 65.49*** | 50.05 |
|  | Alpha | 76.23*** | 76.07*** | 49.95 |  | Alpha | 75.74*** | 74.59*** | 47.89 |
|  | Beta | 64.26*** | 57.87*** | 48.80 |  | Beta | 63.11*** | 59.84*** | 48.59 |
|  | Broadband | 69.75*** | 64.92*** | 50.79 |  | Broadband | 67.38*** | 63.93*** | 50.90 |
| 3 | Delta | 71.48*** | 70.33*** | 48.37 | 4 | Delta | 70.41*** | 69.43*** | 46.31 |
|  | Theta | 66.39*** | 64.84*** | 49.63 |  | Theta | 65.33*** | 62.54*** | 47.15 |
|  | Alpha | 75.41*** | 74.26*** | 53.45 |  | Alpha | 74.75*** | 73.12*** | 52.24 |
|  | Beta | 62.05*** | 59.10*** | 47.99 |  | Beta | 63.44*** | 56.64*** | 47.13 |
|  | Broadband | 69.59*** | 63.52*** | 48.98 |  | Broadband | 67.21*** | 63.44*** | 49.97 |
| 5 | Delta | 68.28*** | 66.80*** | 49.17 | 6 | Delta | 68.20*** | 66.39*** | 53.13 |
|  | Theta | 65.00*** | 62.05*** | 51.28 |  | Theta | 66.89*** | 63.28*** | 47.71 |
|  | Alpha | 74.02*** | 73.28*** | 49.88 |  | Alpha | 73.44*** | 72.21*** | 50.43 |
|  | Beta | 61.89*** | 58.20*** | 48.43 |  | Beta | 66.97*** | 64.18*** | 51.12 |
|  | Broadband | 67.30*** | 61.72*** | 47.20 |  | Broadband | 65.82*** | 60.08*** | 47.37 |
| 7 | Delta | 66.48*** | 63.44*** | 49.22 | 8 | Delta | 64.75*** | 61.56*** | 50.13 |
|  | Theta | 66.39*** | 62.71*** | 47.56 |  | Theta | 66.39*** | 62.54*** | 52.47 |
|  | Alpha | 71.39*** | 70.08*** | 48.30 |  | Alpha | 70.74*** | 69.26*** | 47.92 |
|  | Beta | 66.31*** | 64.92*** | 49.65 |  | Beta | 66.64*** | 65.41*** | 48.91 |
|  | Broadband | 65.57*** | 60.82*** | 52.90 |  | Broadband | 64.84*** | 62.13*** | 50.24 |
| 9 | Delta | 65.98*** | 62.87*** | 49.47 | 10 | Delta | 65.49*** | 63.36*** | 47.04 |
|  | Theta | 64.92*** | 62.13*** | 50.61 |  | Theta | 64.59*** | 62.05*** | 50.33 |
|  | Alpha | 70.25*** | 68.44*** | 49.80 |  | Alpha | 70.08*** | 68.36*** | 51.33 |
|  | Beta | 66.64*** | 64.26*** | 51.27 |  | Beta | 66.23*** | 64.02*** | 52.61 |
|  | Broadband | 64.51*** | 61.48*** | 49.63 |  | Broadband | 64.51*** | 61.48*** | 49.22 |
| 11 | Delta | 65.33*** | 62.71*** | 48.63 | 12 | Delta | 64.67*** | 61.97*** | 49.01 |
|  | Theta | 64.26*** | 60.08*** | 49.70 |  | Theta | 64.51*** | 60.33*** | 51.22 |
|  | Alpha | 70.33*** | 67.46*** | 47.78 |  | Alpha | 68.61*** | 66.31*** | 49.62 |
|  | Beta | 66.48*** | 63.69*** | 52.36 |  | Beta | 65.00*** | 63.61*** | 50.09 |
|  | Broadband | 65.08*** | 62.05*** | 51.18 |  | Broadband | 65.00*** | 61.48*** | 51.62 |
| 13 | Delta | 65.00*** | 62.62*** | 49.06 | 14 | Delta | 64.18*** | 62.37*** | 47.14 |
|  | Theta | 63.44*** | 60.00*** | 48.95 |  | Theta | 63.77*** | 60.82*** | 49.04 |
|  | Alpha | 68.53*** | 66.72*** | 47.84 |  | Alpha | 68.52*** | 66.56*** | 47.90 |
|  | Beta | 65.41*** | 64.18*** | 48.77 |  | Beta | 64.51*** | 61.97*** | 48.93 |
|  | Broadband | 64.02*** | 61.48*** | 51.31 |  | Broadband | 64.75*** | 62.46*** | 50.89 |
| 15 | Delta | 63.93*** | 62.30*** | 49.90 |  |  |  |  |  |
|  | Theta | 63.36*** | 60.49*** | 48.42 |  |  |  |  |  |
|  | Alpha | 68.61*** | 66.48*** | 50.27 |  |  |  |  |  |
|  | Beta | 64.84*** | 61.48*** | 48.58 |  |  |  |  |  |
|  | Broadband | 62.79*** | 60.25*** | 48.01 |  |  |  |  |  |

*In the above table, balanced and 10 x 10 cross-validation accuracies for each frequency-specific model are provided. These values were averaged over ten runs and across all the possible numbers of features (k = 1:15). Using a paired sample t-test, each accuracy was compared to its corresponding null (surrogate) accuracy. Asterisks indicate significance (* p < 0.05, ** p < 0.005, *** p < 0.0005).*
